# Human Papillomaviruses and genital co-infections in gynaecological outpatients

**DOI:** 10.1186/1471-2334-9-16

**Published:** 2009-02-12

**Authors:** Rosita Verteramo, Alessandra Pierangeli, Emanuela Mancini, Ettore Calzolari, Mauro Bucci, John Osborn, Rosa Nicosia, Fernanda Chiarini, Guido Antonelli, Anna Marta Degener

**Affiliations:** 1 Department of Gynaecology, Perinatology and Child Health, "Sapienza" University of Rome, Italy; 2 Department of Experimental Medicine, Section of Virology, "Sapienza" University of Rome, Italy; 3 Department of Public Health, "Sapienza" University of Rome, Italy; 4 Department of Public Health, Section of Microbiology, "Sapienza" University of Rome, Italy

## Abstract

**Background:**

High grade HPV infections and persistence are the strongest risk factors for cervical cancer. Nevertheless other genital microorganisms may be involved in the progression of HPV associated lesions.

**Methods:**

Cervical samples were collected to search for human Papillomavirus (HPV), bacteria and yeast infections in gynaecologic outpatients. HPV typing was carried out by PCR and sequencing on cervical brush specimens. *Chlamydia trachomatis *was identified by strand displacement amplification (SDA) and the other microorganisms were detected by conventional methods.

**Results:**

In this cross-sectional study on 857 enrolled outpatients, statistical analyses revealed a significant association of HPV with *C. trachomatis *and *Ureaplasma urealyticum (*at high density) detection, whereas no correlation was found between HPV infection and bacterial vaginosis, *Streptococcus agalactiae*, yeasts, *Trichomonas vaginalis *and *U. urealyticum*. *Mycoplasma hominis *was isolated only in a few cases both in HPV positive and negative women and no patient was infected with *Neisseria gonorrhoeae*.

**Conclusion:**

Although bacterial vaginosis was not significantly associated with HPV, it was more common among the HPV positive women. A significant association between HPV and *C. trachomatis *was found and interestingly also with *U. urealyticum *but only at a high colonization rate. These data suggest that it may be important to screen for the simultaneous presence of different microorganisms which may have synergistic pathological effects.

## Background

Infection with high grade Human papillomavirus (HPV) plays a central role in cervical carcinogenesis. Epidemiological and molecular investigations have shown unequivocally that high grade HPV infection is the main causal factor in initiating the progressive transformation that leads to cervical intraepithelial neoplasia and to cervical cancer [1,2]. Many reports have demonstrated that HPV infections are mostly transitory and that only a small percentage of infections persist and may progress to dysplastic cervical lesions: type-specific HPV persistence is the strongest risk factor for cervical cancer [3]. The reasons for the variable natural history remain poorly understood but it has been generally accepted that different cofactors are involved in the development of dysplasia in HPV infected women. Factors, such as specific HPV genotype, viral load, and co-infection with more HPV types, are likely to be involved in the progression to precancerous cervical lesions [4-6]. In addition, environmental factors [7-9] including smoking, long term hormone contraceptives and co-infection with other microorganisms have also been investigated as potentially involved in the disease process. The sexually transmitted agents that have shown some evidence of an association with cervical dysplastic and neoplastic lesion are *Chlamydia trachomatis *[10] and Herpes simplex virus (HSV) [11,12], although the specific role of these microorganisms in the pathogenesis of cervical cancer has not been completely clarified.

The aim of this cross-sectional study was to evaluate the prevalence of co-infection HPV and other genital microorganisms: *Chlamydia trachomatis*, *Neisseria gonorrhoeae*, *Streptococcus agalactiae, Mycoplasma spp, Trichomonas vaginalis*. Yeasts and bacterial vaginosis were also investigated.

## Methods

### Study population

Women attending clinics for routine gynaecologic care in the Department of Gynaecology, Perinatology and Child Health of "Sapienza" University in Rome, between 2000 and 2006 were included in this study. Informed consent was obtained from all women and this study was approved by the Ethics Committees and/or Institutional Review Boards of the participating institution. Cervical samples were taken by cytobrush from the ectocervix and from the endocervix for cytological analysis, detection of HPV DNA and bacteriological tests. Cytology was classified according to the Bethesda system, using standard forms and classified as negative, atypical squamous cell of undetermined significance (ASCUS), atypical glandular cell of undetermined significance (AGUS), low-grade intraepithelial lesion (LSIL) or high-grade (HSIL) categories [13]. All the samples were analysed independently by two cyto-technologists and the final diagnosis was based on the consensus of both. Women were eligible for the study if they: were not currently pregnant nor planning to become pregnant for a year; had an intact uterus and no current referral for hysterectomy; reported no use of vaginal medication in the previous 3 days; reported no treatment for cervical disease in the previous 6 months; answered a structured questionnaire on socio-demographic characteristics, sexual behaviour, reproductive history and smoking habits; provided samples adequate for PCR assays and sequencing. According to these criteria, 857 women were enrolled in this cross-sectional study.

### HPV detection and typing

Cervical brush samples collected in Phosphate Saline Buffer (PBS) were centrifuged at low speed and the cell pellets underwent DNA extraction using QIAampTissue kit (Qiagen, Milan-Italy). Human Leukocyte Antigen (HLA)-specific primers were used in polymerase chain reaction (PCR) analysis to assess the suitability of the target DNA (9). A 450 bp fragment from the L1 region of HPV-DNA was amplified using the consensus primer MY09/11 as described elsewhere [14]. Four pairs of degenerated consensus primers (able to detect 36 HPV types) were used concomitantly to amplify the complete *E6 *gene and part of the *E7 *gene [15], thus allowing the detection of a broader range of HPV genotypes. The PCR products were detected by ethidium bromide staining after electrophoretic migration through 2% agarose gels.

HPV-positive reactions were purified using a QIAquick PCR purification kit (QIAGEN), and an automatic DNA sequencer (Applied Biosystems, Foster City, CA) was used for sequencing utilising the PCR forward primer(s). Sequence homology was determined using BLAST (NCBI of NIH, Bethesda, MD) and Clustal W (EMBL-EBI, Heidelberg-Germany) programs.

### Detection of microorganisms

#### Streptococcus agalactiae

*Streptococcus agalactiae *(group B) strains were identified from clinical samples by being weakly beta-haemolytic on Columbia CNA Agar A (Oxoid). The suspected colonies were serologically confirmed (Lancefield's classification) [16,17].

#### Chlamydia trachomatis

endocervical swabs were tested for the presence of *C. trachomatis *using the BD ProbeTec ET Assays (BDPT, Becton Dickinson and Company, Franklin Lakes, NJ) according to the manufacturer's instructions. This method amplifies DNA from *C. trachomatis *in separate wells and optionally can monitor inhibition of amplification for each specimen using strand displacement amplification (SDA) and detection by fluorescent energy transfer (FRET) probes, producing a method-other-than-acceleration (MOTA) score for each specimen. Specimen processing and BDPT performance followed the manufacturer's instructions. The original algorithm involved retesting specimens with MOTA scores between 2000 and 9999. A negative repeat result (MOTA score <2000) was considered indeterminate.

#### Neisseria gonorrhoeae

presumptive identification of *N. gonorrhoeae *was carried out by growth on media selective for pathogenic *Neisseria species *(Oxoid) incubated for up to 48 hours in 5–10% CO2 at 35–37°C. Colonies obtained were identified by API NH (bioMerieux) according to manufacturer's instructions.

#### Mycoplasma spp

Mycoplasma IST 2 (bioMérieux) was used for the isolation of *Mycoplasma hominis *and *Ureaplasma urealyticum *according to the manufacturer's instructions. The diagnostic kit provided information regarding the presence or absence of *M. hominis *and *U. urealyticum *and an estimate of the density of each organism (cut-off 10^4^-colour-changing units- CCU/ml) together with its antimicrobial susceptibilities to doxycycline, josamycin, ofloxacin, erythromycin, tetracycline, pristinamycin, azithromycin, clarythromycin, ciprofloxacin.

#### Candida spp

cervical-vaginal samples were cultured on Sabouraud dextrose agar (Oxoid) aerobically at 37°C for 24–48 h [16,17]. The identity of clinical isolates was confirmed by conventional mycological methods [18,19], such as the germ tube induction test in serum (*C. albicans *or *C. dubliniensis*), microscopic morphology, and growth on *Oxoid Chromogenic Candida Agar *(Oxoid) [18].

Non-Candida albicans yeast isolates were further speciated with API 20C AUX or API 32C.

#### Trichomonas vaginalis

the vaginal swab was placed in 0.2 ml of sterile physiologic saline for wet mount evaluation and examined microscopically (400×) within 15 min for motile trichomonads.

The culture was carried out on Oxoid's Trichomonas Medium n.2 for the recovery of *T. vaginalis *and incubated aerobically a 37°C. The broth was examined microscopically for motile trichomonads on days 1, 3 and 5 [16,17].

#### Bacterial vaginosis

the presence of bacterial vaginosis was evaluated microscopically on samples collected from the posterior vaginal fornix. Bacterial vaginosis was diagnosed by the presence of three of the clinical and microscopic findings standardized by Amsel *et al *[20]: vaginal pH greater than 4.5; presence of clue cells; grey homogenous vaginal discharge; positive Whiff-test in which a fishy odour is released after the addition of 10% potassium hydroxide solution to vaginal fluid.

### Statistical analysis

The χ^2 ^test was used in the statistical analysis, while the Odd Ratio was used to measure the strength of the association with HPV. Statistical tests were considered significant if the p value was 0.05 or less. Logistic regression analysis was used to assess the simultaneous effect of more than one variable on the risk of HPV infection and to identify possible confounding factors.

## Results

### Study group characteristics

Out of 857 enrolled outpatients, two groups of patients were identified on the basis of HPV DNA test positivity. The demographic and behavioural characteristics of the study population (266 HPV positive and 591 HPV negative women) are presented in table 1. The women were aged between 17 and 57 years (mean 32.7 years). As expected, the features significantly associated with HPV-DNA detection in univariate analysis were: young patient's age; age at first intercourse; high number of lifetime sexual partners. Smokers were 29.98% in the total population with a significantly higher percentage in HPV positive group (38.1% vs. 28.0; p = 0.003). HPV-DNA detection was significantly higher in the nullipara women and in those without spontaneous abortion. Only 22.40% of the study participants usually use at least one contraceptive method with no significant differences in HPV positivity.

**Table 1:** Characteristics of studied groups: univariate analysis of age, sexual, reproductive and behavioural history.

	HPV(+)	HPV(-)	Total	HPV(+) %	Comparison*	95% CI	significance
TOTAL: frequency	266	591	857	31.0%			

**Age**							
mean age (years) (sd)	31.1(8.0)	33.5(7.5)	32.7(7.6)		diff. -2.4	-1.3 to -3.5	t = -4.26, P < 0.00005
Frequency:							
<25 years	63	72	135	46.7%	OR = 1.00		
25- years	58	116	174	33.3%	OR = 0.57	0.36 to 0.91	P = 0.017
30- years	68	142	210	32.4%	OR = 0.55	0.35 to 0.85	P = 0.008
35- years	36	144	180	20.0%	OR = 0.29	0.17 to 0.47	P < 0.00005
40+ years	41	117	158	25.9%	OR = 0.40	0.25 to 0.65	P = 0.0003
**Age at 1st intercourse**							
mean age (years)	18.6	19.6	19.3		diff. -1.01	-0.49 to -1.54	t = -3.79, P = 0.0002
Frequency:							
<16 years	25	42	67	37.3%	OR = 1.00		
16 years	34	55	89	38.2%	OR = 1.04	0.54 to 2.00	P = 0.91
17 years	44	73	117	37.6%	OR = 1.01	0.54 to 1.88	P = 0.97
18 years	58	115	173	33.5%	OR = 0.85	0.47 to 1.52	P = 0.58
19 years	36	55	91	39.6%	OR = 1.10	0.57 to 2.11	P = 0.77
20 years	21	87	108	19.4%	OR = 0.41	0.20 to 0.81	P = 0.01
21 years	15	24	39	38.5%	OR = 1.05	0.47 to 2.37	P = 0.91
22+ years	33	140	173	19.1%	OR = 0.40	0.21 to 0.74	P = 0.004
**Number of partners**							
mean	4.20	2.83	3.26		diff. 1.37	0.96 to 1.79	t = 6.51, P < 0.00005
frequency:							
1 partner	39	234	273	14.3%	OR = 1.00		
2 partners	46	129	175	26.3%	OR = 2.14	1.33 to 3.45	P < 0.00005
3 partners	58	86	144	40.3%	OR = 4.05	2.52 to 6.51	P < 0.00005
4 partners	41	42	83	49.4%	OR = 5.86	3.39 to 10.13	P < 0.00005
5–9 partners	54	75	129	41.9%	OR = 4.32	2.65 to 7.03	P < 0.00005
10+ partners	28	25	53	52.8%	OR = 6.72	3.55 to 12.71	P < 0.00005
**Parity**							
Nulliparous	215	432		33.2%	OR = 1.00		
Parous	51	159		24.3%	OR = 0.64	0.45 to 0.92	P = 0.0154
Spontaneous abortion							
No	238	485	723	32.9%	OR = 1.00		
Yes	28	106	134	20.9%	OR = 0.54	0.35 to 0.84	P = 0.0063
Induced abortion							
No	227	493	720	31.5%	OR = 1.00		
Yes	39	98	137	28.5%	OR = 0.86	0.58 to1.29	P = 0.48
**Contraceptives**							
never used any type	210	455	665	31.5%	OR = 1.00		
have used at least one type	56	136	192	29.2%	OR = 0.89	0.63 to 1.27	P = 0.52
**Smoking**							
never smoked	168	432	600	28.0%	OR = 1.00		
ever smoked	98	159	257	38.1%	1.58	1.17 to 2.16	P = 0.003

*diff. is the difference between two mean values and OR is the odds ratio compared with the first category.

### HPV genotyping

HPV genotypes found in samples from HPV positive women were shown in table 2. Thirty-five different HPV types were identified: HPV16 was the most prevalent (14.50%), followed by HPV58 (9.16%), HPV53 (8.01%) and HPV42 (6.10%); whereas each other genotype was present in less than 5% of HPV positive women. In addition there were 4 cases of "missed typing" (1.5%), probably due to mixed infections with different HPV genotypes. Moreover it should be considered that PCR amplification and sequencing detect precisely the more abundant (and probably clinically relevant) type and underestimate co-infections. According to the reclassification proposed by Munoz *et al*. (2006) [3], the aggregated percentage (HR and probably HR) of detected HPV was 58.77%, 27.86% for low-risk and 13.35% for unknown-risk HPV types. Of the HPV negative women, 2.3% revealed an abnormal cervical cytology (2% LG-SIL; 0.3% HG-SIL). Among HPV positive women more than 60% had normal cytology, whereas only 3.98% had HG-SIL. LG-SIL and ASCUS were found in 29.8% and 3.1% respectively of HPV positive women.

**Table 2:** Genotypes in HPV positive women.

Risk category*												
**HIGH:**												**total %**
**HPV type**	**16**	**18**	**31**	**33**	**35**	**45**	**51**	**52**	**56**	**58**	**59**	
Frequency	38	7	13	8	2	1	5	8	5	24	3	114 43.51
**Probably HIGH:**												
**HPV type**	**53**	**66**	**68**	**73**								
frequency	21	12	1	6								40 15.27
**LOW:**												
**HPV type**	**6**	**11**	**42**	**43**	**44**	**54**	**61**	**70**	**72**	**81**	**91**	
frequency	13	6	16	1	2	11	6	10	1	5	2	73 27.86
**UNKNOWN:**												
**HPV type**	**30**	**34**	**62**	**67**	**74**	**84**	**85**	**86**	**87**			
frequency	3	3	1	2	5	7	2	2	10			35 13.36

*According to Munoz *et al*. (2006) [3], 11 types were classified as high risk, 4 types as probably high-risk; 11 were classified as low-risk. Nine types were grouped as unknown-risk HPV because of their incomplete characterization due to their rare detection or recent identification.

### Different microorganisms infection rate

In the total population, *C. trachomatis *was revealed in 8.05% of the samples, *U. urealyticum *was present in 28.35% and *S. agalactiae *in 8.86%; *T. vaginalis *was found only in 1.16%, bacterial vaginosis in 6.30%, and yeast in 13.06%.

*M. hominis *was isolated only in a few cases whereas *N. gonorrhoeae *was never found.

### Co-infection HPV-other microorganisms

The detection of genital infections in the HPV negative population was not infrequent: in fact *S. agalactiae *was found in 9.4% of samples, yeasts in 13.5%, bacterial vaginosis in 5.2%, *C. trachomatis *in 5.4% and *U. urealyticum *in 24.8%. Generally *U. urealyticum *is a common commensal of the female lower genital tract and it seems to be an important opportunistic pathogen during pregnancy and in other genital diseases such as cervicitis. Its pathogenic potential seems to be related to the high density in the analysed samples (>10,000 CCU/ml). In HPV negative patients the percentage of *U. urealyticum *> 10,000 CCU/ml was 5.24% (table 3).

**Table 3:** Different microorganisms infection in HPV positive and negative women

	HPV(+)	HPV(-)	Total	% positive	odds ratio	95% CI	Significance
***C. trachomatis***							
negative	229	559	788	29.0%	OR = 1.00		
Positive	37	32	69	53.6%	OR = 2.82	1.74 to 4.57	P < 0.00005
***U. urealyticum***							
negative	170	444	614	27.7%	OR = 1.00		
<10000 CCU/ml	61	116	177	34.5%	OR = 1.37	0.96 to 1.96	P = 0.08
>10000 CCU/ml	35	31	66	53.0%	OR = 2.95	1.79 to 4.85	P < 0.00005
***S. agalactiae***							
negative	246	535	781	31.5%	OR = 1.00		
Positive	20	56	76	26.3%	OR = 0.78	0.46 to 1.32	P = 0.35
**Yeasts**							
negative	234	511	745	31.4%	OR = 1.00		
Positive	32	80	112	28.6%	OR = 0.87	0.56 to 1.35	P = 0.55
**Bacterial vaginosis**							
negative	243	560	803	30.3%	OR = 1.00		
Positive	23	31	54	42.6%	OR = 1.71	0.98 to 2.98	P = 0.06
***T. vaginalis***							
negative	263	584	847	31.1%	OR = 1.00		
Positive	3	7	10	30.0%	OR = 0.95	0.24 to 3.71	P = 0.94

The number of the specimens in which *T. vaginalis *was detected was very low in both populations (1.12% in HPV positive women vs. 1.18%).

Statistical analyses did not reveal any association between HPV presence and *S. agalactiae*, yeasts, *T. vaginalis *and *U. urealyticum *< 10,000 CCU/ml detection.

Although the bacterial vaginosis was diagnosed in a higher percentage among HPV positive women (8.6% vs. 5.2% control group), the difference in the prevalence of bacterial vaginosis was not statistically significant between the 2 groups.

A significant association between HPV-DNA presence and concurrent genital infection with *C. trachomatis *(p < 0.001) was observed (table 3). Interestingly, among HPV positive women co-infected by *C. trachomatis *67.56% belonged to the HR-HPV infected patients.

Moreover another significant association was found between the HPV positivity and the presence of *U. urealyticum *> 10,000 CCU/ml (p < 0.001) (table 3).

Logistic regression analyses did not substantially change these results.

## Discussion

Our findings confirm that an increased risk of HPV infection is strongly associated with a young age at first intercourse, and with an elevated number of sexual partners as would be expected for a sexually transmitted viral infection.

Epidemiological studies have demonstrated the role of other sexually transmitted infectious agents in the pathogenesis of cervical cancer, but knowledge about the specific role of these pathogens in the natural history of HPV infection is limited. There are several mechanisms by which these co-infections may act, such as direct genotoxicity, but probably the most likely biologic mechanism is the induction of cervical inflammation leading genotoxic damage through oxidative metabolites [21]. An association between cervical cancer and *C. trachomatis *has been established [22]. In fact, *C. trachomatis *infection may increase susceptibility to HPV causing microabrasions or alterations of epithelial cells thus facilitating the entry of virions. Some reports have suggested that concurrent *C. trachomatis *infection can reduce host ability to resolve HPV infection: chronic cervical inflammation seems to influence the HPV persistence through a raised production of free radicals and a reduction of host cell-mediated immunity [2,10]. *C. trachomatis *infection induces a shift in the immune response and the unresolved infections have been associated with a humoral (T helper cell type 2) immune response, whereas cellular (T helper cell type 1) immune response is important for the clearance of HPV lesions. Therefore, the modulation of cervical immune response by *C trachomatis *may influence the clearance of HPV lesions [10].

Our data showed, in HPV positive patients, particularly in HR HPV positive women, an increased *C. trachomatis *infection rate; this result may suggest more accurate screening for *C. trachomatis *in HPV positive patients with an atypical cytology. Since both pathogens are co-variables related to sexual behaviour, probably they synergize in inducing cervical epithelium alterations [3,23].

An increased infection rate of *U. urealyticum *in HPV positive group was also observed, but a significant association was found only with high density cervical presence. The biological role of *U. urealyticum *infection in the HPV outcome was not previously clarified, but high level of *U. urealyticum *seems to be a cofactor in the development of HPV related cervical dysplasia [24]. It is tempting to speculate that the presence of *U. urealyticum *may play a role both in initiating cellular anomalies and in viral persistence. It was reported that Mycoplasma infections cause in vitro chromosomal changes and cell transformation throughout gradual progressive chromosomal loss and translocations [25]. Robertsonian chromosome translocation has been identified in the presence of Mycoplasma infections [26].

Previous researches have produced conflicting results regarding the role of bacterial vaginosis as cofactor in the development of cervical intraepithelial neoplasia [27]. Thus, although bacterial vaginosis may have an influence in acquisition of genital infections, its role into HPV acquisition and persistence remains to be clarified [28]. Moreover, to date the only prospective study on bacterial vaginosis and HPV infection, showed that both infections occur simultaneously [29] and suggested that the presence of HPV may have an influence in the vaginal flora. In our findings, bacterial vaginosis is not statistically associated with HPV infection, even if alterations of vaginal flora tend to be more common among HPV positive than HPV negative women (8.6% vs. 5.2% respectively).

This study evidenced also the lack of protective precautions against sexually transmitted infections, both in HPV positive and HPV negative women. Despite the use of condom is not reported to be highly effective in reducing HPV transmission [30,31], counselling on sexually transmitted diseases (STDs) prevention use should be continued, for instance, to prevent *C. trachomatis *transmission.

## Conclusion

In this study a statistically significant association between HPV and *C. trachomatis *was found and interestingly also with *U. urealyticum *at a high colonization rate. Although bacterial vaginosis was not significantly associated with HPV, it was more common among HPV positive women. These data confirm that screening for genital infections may be important to reveal the simultaneous presence of different sexually transmitted microorganisms. These results suggest and emphasize the value of the screening for genital infections in HPV positive patients in order to decrease the presence of other microorganisms and to reduce the probable synergistic effects of co-infections. Prevention is important not only to avoid other STDs and their sequelae, but also to reduce the influence of concomitant microorganisms on HPV infection.

## Competing interests

The authors declare that they have no competing interests.

## Authors' contributions

RV, AP, EC and AMD conceived the study, collected and interpreted the data and wrote the manuscript; EM, EC and RV collected the samples and analysed the clinical data; JO performed the statistical analyses and drafted the manuscript; RN and FC carried out the bacterial analyses and contribute to draft the manuscript; MB performed the HPV DNA tests and participated in the sequence analysis with AP and AMD; GA participated to the design of the study and was involved in drafting and revising critically the manuscript; All authors read and approved the final manuscript.

## Pre-publication history

The pre-publication history for this paper can be accessed here:

http://www.biomedcentral.com/1471-2334/9/16/prepub
